# Structured tapering of oral corticosteroids in patients with severe asthma at risk of secondary adrenal insufficiency: a case series

**DOI:** 10.11604/pamj.2025.52.44.48421

**Published:** 2025-09-29

**Authors:** Manuel Carpio Salmerón, Adrian Heredia Carrillo, Luis Marin Martinez, Georgios Kyriakos

**Affiliations:** 1 Servicio de Endocrinología y Nutrición, Hospital General Universitario Santa Lucía, Cartagena, España,; 2 Servicio de Neumología, Hospital General Universitario Santa Lucía, Cartagena, España

**Keywords:** Severe asthma, corticosteroids, secondary adrenal insufficiency, biologics

## Abstract

Severe asthma is a chronic condition that often requires oral corticosteroid (OCS) therapy, which, when prolonged, may lead to significant adverse effects, including secondary adrenal insufficiency. Advances in biologic therapies have allowed many patients to reduce or discontinue OCS. We describe the clinical course and outcomes of six patients with severe corticosteroid-dependent asthma who underwent a structured OCS tapering protocol under endocrinological supervision. All patients had a history of prolonged OCS use (mean duration >10 years), high-dose inhaled corticosteroids (ICS), and biologic therapy. Adrenal insufficiency was confirmed in three patients (50%), who required continued hydrocortisone replacement due to persistent hypothalamic-pituitary-adrenal (HPA) axis suppression. The other three patients successfully discontinued corticosteroids and demonstrated HPA axis recovery. Notably, no asthma exacerbations occurred during the tapering process. A structured, multidisciplinary corticosteroid tapering protocol is both feasible and safe in patients with severe asthma, particularly when guided by endocrine assessment. ICS exposure may contribute to adrenal suppression, highlighting the need for comprehensive hormonal evaluation in this population.

## Introduction

Severe asthma affects approximately 3-10% of all asthma patients, yet it accounts for a disproportionate burden in terms of morbidity, healthcare resource utilization, and reduced quality of life [1]. It is defined as asthma that requires high-dose inhaled corticosteroids plus a second controller (typically a long-acting β_2_-agonist), or systemic corticosteroids for most of the year to maintain control, or remains uncontrolled despite such treatment [2]. In many of these patients, chronic use of systemic corticosteroids, although effective in controlling exacerbations and persistent symptoms, is associated with numerous well-documented adverse effects. These include hypertension, osteoporosis, steroid-induced diabetes, cataracts, secondary adrenal insufficiency, and increased risk of severe infections [3,4].

Recent advances in asthma treatment, particularly the introduction of biologic therapies, have significantly reduced the reliance on systemic corticosteroids in selected patients [5-7]. This paradigm shift underscores the need for structured corticosteroid tapering strategies. These aim to minimize the risks associated with long-term corticosteroid use, particularly adrenal insufficiency, and to improve treatment safety [8]. International guidelines, such as GINA (Global Initiative for Asthma), recommend initiating systemic corticosteroid reduction when biologic therapy is started or when sustained disease control is achieved, under close medical supervision.

Adrenal insufficiency is a condition in which the adrenal glands fail to produce adequate amounts of hormones, primarily cortisol. It is classified as primary, resulting from intrinsic adrenal pathology, or secondary, due to suppression of the hypothalamic-pituitary-adrenal (HPA) axis, often caused by prolonged exposure to exogenous glucocorticoids [9,10]. In patients with severe asthma, chronic use of oral and inhaled corticosteroids can lead to sustained suppression of endogenous cortisol production, resulting in secondary adrenal insufficiency. Patients with severe asthma treated with systemic corticosteroids (SCS) are at elevated risk of HPA axis suppression and, consequently, more susceptible to adrenal crisis following corticosteroid withdrawal. These high-risk groups are summarized in Table 1. The objective of this study is to describe a case series of patients with controlled severe asthma in whom a structured oral corticosteroid tapering protocol was implemented, in accordance with the GEMA 5.2 guidelines [11] (Table 2).

**Table 1:** clinical criteria for high risk of hypothalamic-pituitary-adrenal (HPA) axis suppression in patients with asthma [19]

Risk category	Clinical criteria-definition
Repeated systemic corticosteroid use	≥ 4 courses per year or ≥ 30 cumulative days of treatment per year
Daily high-dose inhaled corticosteroids	e.g., fluticasone >1000 µg/day or beclomethasone >800 µg/day
Prolonged systemic corticosteroid therapy	Daily or alternate-day use for more than 3 months
Combined ICS and systemic corticosteroids	Concurrent use of medium or high dose ICS with systemic corticosteroids.
Clinical suspicion of adrenal suppression	Symptoms such as fatigue, hypotension or hyponatremia during corticosteroid tapering.

**Table 2:** structured oral corticosteroid tapering protocol for patients with severe asthma [11]

Gradually reduce the initial oral corticosteroid (OCS) dose by approximately 10% per week until a physiological dose is reached (equivalent to 15-30 mg/day of hydrocortisone)
Once the physiological threshold is achieved, substitute OCS with an equivalent dose of hydrocortisone (e.g. 20 mg/day, administered in 2-3 divided doses) for approximately 10 days.
If well tolerated, reduce the hydrocortisone dose by 50% and maintain this dose additional 10 days.
**Assess adrenal function:**
If morning serum cortisol >12 µg /dL → recovery of the HPA axis can be assumed.
If 3-12 µg/dL → perform ACTH stimulation test.
If <3 µg/dL → persistent adrenal insufficiency is likely; continue replacement therapy.

## Methods

**Study design:** a longitudinal case series study was conducted involving patients diagnosed with controlled severe asthma who had initiated biologic therapy and were referred from the Pulmonology Department to the Endocrinology Department for the implementation of a systemic glucocorticoid tapering protocol.

**Study setting:** this longitudinal case series was conducted at the Endocrinology and Nutrition Department of the *Hospital General Universitario Santa Lucía*, located in Cartagena, Murcia, Spain. The study population consisted of six adult patients (aged 38 to 75 years) diagnosed with severe corticosteroid-dependent asthma, all of whom had been referred from the Pulmonology Department after initiating biologic therapy.

Patient recruitment occurred prospectively, following referral for the implementation of a structured oral corticosteroid tapering protocol in line with the GEMA 5.2 guidelines. The tapering was conducted under endocrinological supervision, and included systematic assessment of adrenal function before and after corticosteroid reduction. Given the nature of a case series, no formal follow-up period was predefined beyond the tapering process itself. However, patients were monitored clinically throughout the dose reduction, with special attention to symptoms of adrenal insufficiency and asthma control. Hormonal testing included morning serum cortisol and, when needed, ACTH stimulation tests to evaluate recovery of the hypothalamic-pituitary-adrenal axis. The study took place in a real-world clinical setting, using a multidisciplinary approach involving pulmonologists and endocrinologists. All six patients were receiving biologic agents (benralizumab, mepolizumab, or tezepelumab) during the tapering period.

**Participants:** six patients with persistent, corticosteroid-dependent severe asthma were included, ranging in age from 38 to 75 years. The cohort consisted of t hree women and three men. The predominant asthma phenotype was T2 eosinophilic asthma (4 out of 6), with two cases showing a confirmed allergic component and two without allergic sensitization. The remaining two patients had severe intrinsic asthma without an identifiable allergic component.

All patients had received long-term oral glucocorticoid (OGC) therapy, with an estimated mean duration of over 10 years. Three patients had been treated with medium-to-high doses of prednisone (≥10mg/day) for more than a decade, while the remaining three were on low doses (≤5mg/day) at the time of endocrine evaluation. The OGCs used included prednisone, deflazacort, and methylprednisolone. In all cases, high-potency inhaled corticosteroids (beclometasone, fluticasone, budesonide, or mometasone) had been maintained since the initial diagnosis of severe asthma.

Each patient followed a progressive systemic corticosteroid tapering protocol based on the GEMA 5.2 guidelines (Table 2), tailored to the individual clinical scenario. The goal was to achieve complete discontinuation of systemic corticosteroids when feasible. The tapering schedule was adapted based on baseline OGC dose, asthma control status, symptoms suggestive of adrenal insufficiency, and the results of adrenal function testing (including basal cortisol and, when required, ACTH stimulation tests). Basal cortisol was measured at the beginning of the protocol and again after switching to hydrocortisone. Recovery of the hypothalamic-pituitary-adrenal (HPA) axis was defined as a basal cortisol level >12 µg/dL [11].

**Variables:** age, sex, and duration of oral glucocorticoid (OGC) therapy (in years); baseline OGC dose; baseline cortisol level (µg/dL); post-hydrocortisone cortisol level; recovery of the hypothalamic-pituitary-adrenal (HPA) axis (defined as basal cortisol >12 µg/dL); type of biologic therapy received (benralizumab, mepolizumab, or tezepelumab); presence of corticosteroid-related adverse effects and occurrence of asthma exacerbations during the tapering process.

**Data sources:** clinical and biochemical data were extracted from the patients' electronic medical records and hospital endocrine documentation. Hormonal assessments (basal and post-hydrocortisone cortisol levels) were performed at the Clinical Laboratory of *Hospital General Universitario Santa Lucía*, using validated automated immunochemiluminescence assays. When indicated, standard ACTH stimulation tests were conducted to confirm adrenal insufficiency.

**Bias:** the main potential bias was selection bias, as patients were referred from the Pulmonology Department during a period of stable asthma control and ongoing biologic therapy, which may limit the generalizability of findings to the broader population with severe asthma. Additionally, as a case series without a control group, there is a risk of information bias and confounding by unmeasured variables, such as cumulative inhaled corticosteroid exposure. Classification bias was minimized by using a standardized tapering protocol and centralized endocrine evaluation.

**Study size:** a total of six consecutive patients with controlled severe asthma, ongoing biologic therapy, and chronic oral corticosteroid dependence were included. No formal sample size calculation was performed, given the exploratory and descriptive nature of the study.

**Quantitative variables:** age; duration of OGC therapy (years) and basal and post-hydrocortisone cortisol levels (µg/dL). These variables were summarized as means and ranges, and interpreted according to endocrinological thresholds used to define HPA axis recovery.

**Statistical methods:** given the small sample size and case series design, the analysis was purely descriptive. Quantitative variables were reported as mean and range, while qualitative variables (e.g., HPA axis recovery, adverse effects, exacerbations) were expressed as absolute frequencies and percentages. No inferential statistical tests or multivariate analyses were applied. Data were presented in tables to facilitate patient-level comparison.

## Results

A total of six patients with a confirmed diagnosis of severe corticosteroid-dependent asthma, all undergoing biologic therapy, were referred from the Pulmonology Department to the Endocrinology Department for the implementation of a structured oral corticosteroid tapering protocol. All six were evaluated for eligibility and included in the study, with no losses to follow-up during the tapering period.

There were no cases of non-participation or patient exclusion, as all referred individuals met inclusion criteria and consented to participate. Given the small sample size, a flow diagram was not deemed necessary. After referral to the Endocrinology Department, the oral glucocorticoid (OGC) dose was successfully reduced to physiological levels in all cases (100%), followed by substitution with hydrocortisone. The initial hydrocortisone dose was generally 20mg/day, except in one patient who followed an alternate-day regimen (20mg every 48 hours). All patients tolerated the substitution process without complications.

In terms of complete glucocorticoid withdrawal, three patients (50%) successfully discontinued hydrocortisone and demonstrated recovery of the hypothalamic-pituitary-adrenal (HPA) axis, as indicated by basal cortisol levels >12 μg/dL (Table 3). The remaining three patients did not achieve full recovery of the corticotropic axis and continued on minimal doses of hydrocortisone-except for patient 5-due to persistent iatrogenic secondary adrenal insufficiency, with cortisol levels <2 μg/dL. One patient (patient 5) required an increase in glucocorticoid dosage (prednisone 10mg/day) during tapering due to an episode of lumbosciatica.

**Table 3:** patient characteristics and outcomes of oral corticosteroid tapering

Patient	Sex	Age (years)	Duration of OCS use (years)	Baseline cortisol (µg/dl)	Cortisol after hydrocortisone switch (µg/dl)	HPA axis recovery
1	Female	38	10	1.6	1.9	No
2	Male	61	8	0.4	1.9	No
3	Male	75	15	7.9	12.6	Yes
4	Male	52	10	8	16	Yes
5*	Female	64	30	11	0.8*	No
6	Female	46	10	8.1	12.6	Yes

* In patient 5, post-hydrocortisone cortisol could not be assessed due to the reintroduction of systemic corticosteroids prior to completion of the tapering protocol.

Notably, none of the patients experienced asthma exacerbations during the tapering process. All were receiving biologic therapy at the time of the endocrinological intervention, including benralizumab (n=2), mepolizumab (n=2), and tezepelumab (n=2). The most frequent adverse effects associated with long-term OGC use included osteopenia and/or osteoporosis in three patients (50%), arterial hypertension in two (33.3%), and obesity with metabolic syndrome in two others (33.3%). Additionally, one patient (16.7%) developed steroid-induced diabetes, and another exhibited clinical features of exogenous Cushing's syndrome (16.7%).

## Discussion

In this small case series, all patients with severe corticosteroid-dependent asthma successfully tapered their prednisone (or equivalent) doses to physiological levels, and half (3 out of 6) achieved complete withdrawal of systemic corticosteroids with restoration of hypothalamic-pituitary-adrenal (HPA) axis function. Notably, in one patient (patient 5), tapering was interrupted due to an unrelated condition-lumbar radiculopathy-which required reintroduction of systemic corticosteroids at higher doses. Given that this patient had baseline cortisol levels nearly compatible with preserved adrenal function (Table 3), it is reasonable to assume that successful withdrawal might also have been achieved in the absence of this confounding factor.

Persistent adrenal insufficiency in the two other non-recovered patients is consistent with existing literature, which has identified several predictors of incomplete HPA axis recovery. First, long-term exposure to medium-to-high doses of oral glucocorticoids (≥10mg/day for more than a decade) is associated with irreversible adrenal atrophy in some cases. Second, chronic use of high-potency inhaled corticosteroids (ICS), such as fluticasone or beclomethasone, may contribute to axis suppression due to their elevated systemic bioavailability. This phenomenon has been documented in both pediatric and adult populations [12,13].

**Limitations:** this study has several limitations. First, the small sample size and descriptive design limit the generalizability of the findings. Additionally, the absence of a control group and the single-center design further constrain the applicability of our results to broader populations. Moreover, the lack of long-term follow-up precludes more definitive conclusions regarding sustained adrenal recovery or long-term safety. However, the homogeneity of the tapering protocol, the absence of asthma exacerbations during follow-up, and the rigorous endocrinological assessment add clinical value to the analysis and support the relevance of a structured glucocorticoid withdrawal approach in selected patients.

Our findings are in line with results from landmark clinical trials such as ZONDA and SIRIUS, in which complete withdrawal of oral corticosteroids was achieved in only 54-56% of participants. In the ZONDA trial, benralizumab significantly reduced oral corticosteroid use over a 28-week period compared to placebo [14]. Similarly, the SIRIUS trial demonstrated that mepolizumab enabled effective tapering of oral corticosteroids following a structured optimization phase [15]. A substantial proportion of patients in both studies required maintenance therapy with low-dose corticosteroids or hormonal replacement due to persistent adrenal insufficiency.

Additional factors such as interindividual variability in response to biologic therapies and genetic polymorphisms in the glucocorticoid receptor may also influence HPA axis recovery. However, these biomarkers are not yet routinely assessed in clinical practice [16-18]. A particularly notable finding in our series is that none of the patients experienced asthma exacerbations during the tapering process. This reinforces the efficacy and safety of biologic agents-benralizumab, mepolizumab, and tezepelumab-as effective corticosteroid-sparing therapies. These outcomes are consistent with those of the PONENTE study, in which 63% of patients discontinued oral corticosteroids without loss of asthma control and with an annualized exacerbation rate of 0.63 [19].

Regarding adverse effects associated with long-term oral corticosteroid use, the most frequent complications were osteoarticular. Osteopenia and/or osteoporosis were present in 50% of the patients, which aligns with the well-established pathophysiology of glucocorticoid-induced osteoporosis. This includes inhibition of bone formation (via osteoblast and osteocyte apoptosis), enhanced bone resorption (via RANK-L-mediated osteoclast activation), and impaired calcium homeostasis. The prevalence observed in our series is comparable to that reported in population-based studies, such as the one by Waljee *et al.*, which demonstrated increased fracture risk even after short-term corticosteroid use [3].

An often underrecognized but clinically relevant aspect emerging from this cohort is the role of inhaled corticosteroids in adrenal suppression. All patients had received high-potency ICS for prolonged periods (e.g., beclomethasone ≥800 µg/day or fluticasone >2000 µg/day), which can lead to partial or complete HPA axis suppression-particularly when combined with systemic corticosteroids. Although less well documented than suppression due to oral corticosteroids, this phenomenon is gaining attention in recent literature and highlights the importance of evaluating ICS doses during corticosteroid tapering protocols [19].

## Conclusion

Prolonged use of high-dose oral corticosteroids in patients with severe asthma frequently results in suppression of the HPA axis and the development of secondary adrenal insufficiency. Our experience demonstrates that a structured, multidisciplinary protocol for oral corticosteroid tapering-guided by systematic endocrinological assessment and supported by biologic therapies-can be implemented safely and effectively in this high-risk population. A substantial proportion of patients achieved complete withdrawal of systemic corticosteroids and recovery of adrenal function, with no asthma exacerbations observed during the tapering process. These findings highlight the importance of individualized tapering strategies and close endocrine monitoring to minimize the risks associated with long-term corticosteroid therapy. Further prospective studies with larger cohorts and extended follow-up are warranted to confirm these results and to optimize tapering protocols across diverse clinical settings.

### What is known about this topic

- Chronic administration of oral corticosteroids induces secondary adrenal insufficiency;
- Monoclonal antibodies reduce corticosteroid requirements in corticosteroid-dependent patients with severe asthma.

### What this study adds

- Using individualized corticosteroid tapering protocols, it is possible, in certain cases, to achieve recovery of the HPA axis;
- High-potency inhaled corticosteroids, when administered at elevated doses, induce inhibition of the HPA axis.

## References

[1] Wenzel SE (2012). Asthma phenotypes: the evolution from clinical to molecular approaches. Nat Med.

[2] Global Initiative for Asthma (GINA) Global Strategy for Asthma Management and Prevention 2024 Update.

[3] Waljee AK, Rogers MA, Lin P, Singal AG, Stein JD, Marks RM (2017). Short term use of oral corticosteroids and related harms among adults in the United States: population based cohort study. BMJ.

[4] Barnes PJ (2013). Corticosteroid resistance in patients with asthma and chronic obstructive pulmonary disease. J Allergy Clin Immunol.

[5] Bel EH, Wenzel SE, Thompson PJ, Prazma CM, Keene ON, Yancey SW (2014). Oral glucocorticoid-sparing effect of mepolizumab in eosinophilic asthma. N Engl J Med.

[6] FitzGerald JM, Bleecker ER, Nair P, Korn S, Ohta K, Lommatzsch M (2016). Benralizumab, an anti-interleukin-5 receptor monoclonal antibody, as add-on treatment for patients with severe, uncontrolled, eosinophilic asthma (CALIMA). Lancet.

[7] Rabe KF, Nair P, Brusselle G, Maspero J, Castro M, Sher L (2018). Efficacy and safety of dupilumab in glucocorticoid-dependent severe asthma. N Engl J Med.

[8] Heaney LG, Busby J, Bradding P, Chaudhuri R, Mansur AH, Niven RM (2019). Biomarkers of treatment response in severe asthma: a systematic review. Clin Exp Allergy.

[9] Zaman N, Hassan M, Ahmed S (2015). Secondary Adrenal Insufficiency: A Review of Clinical Features, Diagnosis and Management. BIRDEM Med J.

[10] Zuechner M, Leelarathna L (2021). Steroid-induced adrenal insufficiency: a hidden danger. BMJ.

[11] Menzies-Gow A, Modificado de: Menzies-Gow (2022). 2022 En: GEMA 5.2. Guía Española para el Manejo del Asma.

[12] Todd GRG, Acerini CL, Ross-Russell R, Zahra S, Warner JT, McCance D (1996). Suppression of the hypothalamic-pituitary-adrenal axis in asthmatic children treated with high-dose fluticasone propionate. Lancet.

[13] Gulliver T, Eid N, Spier S (2020). Adrenal suppression from inhaled corticosteroids: clinical presentation and diagnostic evaluation. Ann Allergy Asthma Immunol.

[14] Ortega HG, Liu MC, Pavord ID, Brusselle GG, FitzGerald JM, Chetta A (2014). Mepolizumab treatment in patients with severe eosinophilic asthma. N Engl J Med.

[15] Charmandari E, Nicolaides NC, Chrousos GP (2014). Adrenal insufficiency. Lancet.

[16] Joseph RM, Hunter AL, Ray DW, Dixon WG (2016). Systemic glucocorticoid therapy and adrenal insufficiency in adults: a systematic review. Semin Arthritis Rheum.

[17] Vogelmeier CF, Asciuto G, Bjermer L, Bourdin A, Dorow P, Fielding S (2020). Clinical implications of corticosteroid-related adverse events in asthma and COPD. Respir Med.

[18] Menzies-Gow A, Gurnell M, Heaney LG, Corren J, Bel EH, Maspero J (2022). Oral corticosteroid elimination via a personalised reduction algorithm in adults with severe, eosinophilic asthma treated with benralizumab (PONENTE): a multicentre, open-label, single-arm study. Lancet Respir Med.

[19] Pérez Conesa M (2013). Guía práctica para el uso de corticoides y terapias inmunosupresoras.

